# Analysis of Training Behavior in Users of a Fitness App: Cross-Sectional Study

**DOI:** 10.2196/72201

**Published:** 2026-01-08

**Authors:** Andrea Fuente-Vidal, Roger Prat, Juan Manuel Arribas-Marin, Oscar Bastidas-Jossa, Myriam Guerra-Balic, Begonya Garcia-Zapirain, Joel Montane, Javier Jerez-Roig

**Affiliations:** 1 Faculty of Psychology, Education and Sports Sciences (FPCEE) Blanquerna Universitat Ramon Llull Barcelona Spain; 2 Research Group on Methodology, Methods, Models and Outcomes of Health and Social Sciences (M3O), Faculty of Health Sciences and Welfare, Centre for Health and Social Care Research (CESS) Universitat de Vic - Universitat Central de Catalunya Vic Spain; 3 San Juan de Dios University School of Nursing and Physical Therapy Comillas Pontifical University Madrid Spain; 4 San Juan de Dios Foundation Madrid Spain; 5 eVIDA Research Group Universidad de Deusto Bilbao Spain; 6 School of Health Sciences, Blanquerna Universitat Ramon Llull Barcelona Spain; 7 Institute of Sport Science and Innovations Lithuanian Sports University Kaunas Lithuania; 8 Institute for Research and Innovation in Life and Health Sciences in Central Catalonia (IRIS-CC) Universitat de Vic - Universitat Central de Catalunya Vic Spain

**Keywords:** fitness app, physical activity, exercise adherence, retention, motivation, mHealth

## Abstract

**Background:**

Mobile health (mHealth) apps are increasingly being used to promote physical activity (PA) and can support exercise uptake and maintenance. Despite their potential, these tools face high dropout rates and inconsistent adherence, posing a significant challenge. Understanding how users engage with fitness apps is essential for improving user experience and health outcomes.

**Objective:**

This study aims to analyze user behavior patterns in the Mammoth Hunters (MH) fitness app (Mammoth Hunters SL), focusing on retention (days from registration to user’s last recorded training session), average weekly training frequency, and adherence (alignment between planned and actual training). We examined how these outcomes are influenced by sociodemographic, motivational, and other variables.

**Methods:**

This cross-sectional study involved 2771 Mammoth Hunters app users. In a subsample (n=289), training data were complemented by motivational data acquired through online surveying via an ad-hoc scale (internal consistency >0.83) based on the self-determination theory (SDT). Descriptive statistics and nonparametric tests (Kruskal-Wallis, Dunn post-hoc, and Spearman correlation) were used to assess correlation between sociodemographic, motivation, and training behavior variables.

**Results:**

Mean retention (days) was significantly higher among males than females (135 vs 109, respectively; *P<*.01), users in the subscription vs free plan (154 vs 81; *P*<.001), active or very active individuals vs inactive, midbuilt vs thin body types (132 vs 120; *P*=.001), and those with slightly lower BMI. Users pursuing antiaging or muscle gain goals showed longer retention than those aiming to lose weight (gain: 132, antiaging: 128, lose weight: 116; *P*<.001). Average weekly frequency (sessions per week) of training was statistically significantly different by sex (male: 1.9 vs female: 1.8; *P*=.04), body type (thin: 1.96 vs mid: 1.77; *P*=.04), activity level (very active: 2.05 vs inactive: 1.83; *P*=.04), and motivation type (extrinsic introjected motivation correlated positively: *r*=0.17; *P*<.05), but did not correlate with perceived difficulty or fitness goals. Adherence, defined as actual vs targeted training frequency, was only significantly different among body types, with thin users showing higher adherence than the midbuilt group (57% vs 52.1%; *P*=.02). Intrinsic motivation showed a positive correlation with retention (*r*=0.19; *P*=.002), as did identified motivation (*r*=0.12; *P*<.05).

**Conclusions:**

This study shows that retention is influenced by demographic factors, with males, subscribers, previously active, midbuilds, those aiming to gain muscle, and individuals with autonomous types (ie, intrinsic and identified) of motivation displaying greater long-term participation. These findings provide valuable preliminary insight into the complexities of exercise training behavior in apps. They suggest that training frequency, retention, and adherence do not respond to the same factors. App developers, researchers, and trainers should assess these variables separately and develop strategies accordingly.

## Introduction

### Adherence to Physical Activity and Exercise

Physical activity (PA) and exercise are fundamental components of a healthy lifestyle, with well-established benefits for physical and mental well-being. PA, as defined by the World Health Organization (WHO), encompasses any bodily movement that results in energy expenditure, while exercise is considered a structured subset of PA, performed with the intent of improving or maintaining physical fitness [1]. Regular engagement in PA is crucial for reducing the risk of chronic diseases, yet adherence to recommended activity levels remains a global challenge [2]. Despite the widespread awareness of PA benefits, sustaining an active lifestyle is often hindered by behavioral, environmental, and psychological barriers [3]. Understanding factors that influence adherence is therefore critical for improving PA participation and ensuring long-term engagement.

### Influence of mobile health on PA Behaviors

In recent years, mobile health (mHealth) apps have emerged as a potential solution to bridge the gap between PA recommendations and actual adherence. Fitness apps, a subset of mHealth, offer structured training programs, progress tracking, and personalized feedback, aiming to enhance motivation and user engagement. The widespread availability of smartphones has contributed to a surge in fitness app usage, with millions of users accessing digital exercise programs globally [4]. These apps incorporate behavior change techniques such as goal setting, social support, and gamification to facilitate sustained exercise habits [5]. However, despite their potential, high attrition rates and inconsistent long-term adherence pose significant challenges to their effectiveness [6]. Recent evidence reinforces that these barriers still persist across different populations and intervention designs. For example, several authors reported significant dropout rates even in gamified or socially incentivized fitness apps [7,8]. Similarly, previous studies highlighted continued adherence challenges in young individuals or older adults despite tailored mHealth interventions [6,7,9].

Adherence to exercise, particularly in digital interventions, remains a complex issue, often inconsistently defined across studies. Traditional adherence models typically assess exercise frequency, duration, and intensity, yet these criteria may not fully capture engagement in app-based fitness programs [3]. Furthermore, users may abandon apps due to technical difficulties, loss of motivation, or unrealistic expectations [10]. Consequently, understanding the determinants of fitness app adherence requires a multidimensional approach, integrating psychological, technological, and behavioral perspectives [11].

The challenges surrounding fitness app adherence are compounded by factors such as user characteristics, app usability, and the broader social and environmental contexts in which users engage with digital interventions. Studies have highlighted that individual attributes such as age, sex, health consciousness, and baseline PA levels may influence the likelihood of sustained engagement with fitness apps [12]. Additionally, app design elements, including intuitive navigation, feedback mechanisms, and interactive features, play a crucial role in user retention [13]. Social and motivational factors, such as competition, social support, and reinforcement strategies, have also been shown to impact adherence levels in digital exercise interventions [5]. Recent evidence highlights the importance of incorporating behavioral theories and enhancing usability and perceived value in reducing attrition and promoting sustained engagement with mHealth tools. For instance, recent findings emphasize the relevance of behavioral theories in crafting more effective mHealth interventions, showing how tailored features can reduce dropout and improve user retention [14]. Similarly, perceived value and usability have been identified as key drivers of long-term engagement with digital health tools [15]. Other studies suggest that personalization, motivational strategies, and social features are critical to increasing user commitment [12,16], overall highlighting the multifactorial nature of adherence and reinforcing the need for user-centered app design approaches.

Given the rising reliance on digital solutions for health and fitness, it is imperative to explore how different aspects of fitness apps contribute to sustained PA engagement. Previous research presents mixed findings on the long-term efficacy of fitness apps in promoting adherence, with some studies reporting positive behavior changes and others limited long-term impact [8,17].

Understanding real users’ training behavior, beyond theoretical frameworks or self-reported intentions, is essential to identify how engagement translates into real-world usage. Analyzing app usage data provides evidence of behavior patterns, allowing researchers to identify which user profiles are more likely to sustain app use. This study establishes a theoretical and practical differentiation between adherence and retention, and how they relate to user motivation to exercise. This is a critical matter, since sustained usage is key to ensuring the long-term impact of digital health interventions [18].

### Study Goal

This study aims to explore the factors influencing the training behavior of users of a fitness app, focusing specifically on exercise adherence, retention, and motivation, and to explore how these outcomes are influenced by sociodemographic, motivational, and training-related factors. We hypothesize that sociodemographic characteristics, motivation types, and training behaviors significantly influence users’ retention, adherence, and frequency of training. Understanding these factors is essential for optimizing fitness app design, improving intervention strategies, and ultimately promoting long-term participation in exercise.

## Methods

### Data Collection and Processing

Data were collected in collaboration with Mammoth Hunters (MH; Mammoth Hunters SL), a fitness app that focused on high-intensity interval exercises to improve strength, endurance, and mobility. MH delivered structured programs rooted in functional movement, making it an ideal platform for investigating digital fitness adherence, motivation, and retention. MH was launched in 2014 by a team of fitness experts and scientists from Barcelona, Spain. A free version with limited access to certain workouts and features was available upon registration, while the Pro version (per subscription) provided full access to personalized plans, a greater variety of workouts, and advanced tracking tools. MH ceased operations in September 2021, being one of the most widely used high-intensity training apps worldwide, having accumulated a total of 719,421 users. The company’s shutdown, as well as its noninvolvement in any of the stages of study, ensured no conflict of interest.

The study used a cross-sectional design. Data in the MH app database included user registries ranging from November 21, 2020, to May 27, 2022. Motivation data were collected via online surveying on March 20, 2022. Data cleaning and descriptive analyses were conducted using the R programming language (version 4.3.1; R Core Team) in the R Studio environment software (version 2023.9.1.494; Posit, PBC).

The initial MH dataset contained 5858 entries, which corresponded to users who had granted informed consent to share their deidentified data for analysis. To ensure the accuracy and relevance of the data, several cleaning steps were executed, including a convenience selection of the most relevant variables and exclusion of registries with insufficient or missing data (see Table S1 in Multimedia Appendix 1 for more detail of the MH app’s original variables).

Outliers were identified through data visualization and consultation of descriptive statistics. A decision was made to remove all outliers to avoid distortion in the analysis, based on two main reasons: (1) certain tests run by MH staff members had intentionally introduced outlier scores to facilitate their identification and removal, and (2) some outliers resulted from the arbitrary temporal cutoff applied to the dataset, specifically, some participants had only just begun training with the app shortly before the data extraction date, leading to unrealistic extreme values in some outcome variables (eg, extremely low retention or extremely high adherence). Therefore, all values exceeding Q3+1.5×IQR or falling below Q1–1.5×IQR were removed.

The motivation-related outcome variables were determined using the means of the composite scores from the observable items corresponding to each factor in the scale, allowing us to define the latent variables. Outcome variables retention, frequency, and adherence were derived from existing variables on the mobile app (eg, number of sessions executed, last training date, and user sign-up date) to enhance analytical depth. User retention was calculated as the number of days between a user's initial registration date within the fitness app and their last recorded training session. Weekly training frequency was calculated by dividing the total number of training sessions completed by a user by the total number of weeks from their first to their last executed session. Adherence was quantified as the percentage to which a user’s actual average weekly training frequency aligned with their initial plan (self-declared upon registration). Additionally, data types were adjusted as required to ensure compatibility and accuracy.

Following the described data cleaning steps, the final dataset comprised 2771 user entries. See Table 1 for more details on study variables.

**Table 1:** Description of explanatory and outcome variables.

Explanatory variables		Type	Description
Sex		Categorical; sociodemographic	User biological sex, with 2 categories: female and male.
Body type		Categorical; sociodemographic	Self-reported body type selected by the user at registration, out of 3 available categories: thin, mid, and strong.
Activity level		Categorical; sociodemographic	User activity level at the time of registration in the fitness app, with 3 categories: inactive, active, and very active.
Fitness goal		Categorical; sociodemographic	The goal the user aims to achieve through app use (selected from 3 available categories: lose weight, gain muscle, and antiaging).
Pro version		Categorical; training	Indicative of app user being subscribed to a payment (“Pro”) program or not. Two categories: yes and no.
Training schedule		Categorical; training	Time of day in which the user executes most (>50%) of their training sessions. Processed into 3 categories: morning (5:30-12:30 hours), afternoon (12:31-20 hours), and night (20-5:29 hours).
Age		Numerical; sociodemographic	User’s reported age at registration.
BMI		Numerical; sociodemographic	User BMI calculated from their declared height and weight.
Subjective body fat		Numerical; sociodemographic	Users’ self-reported body fat.
Difficulty		Numerical; training	Average perceived exertion reported at the end of the training session. 0 (lowest)-10 (highest).
Enjoyment		Numerical; training	Average user-reported enjoyment after each training session. 0 (lowest)-5 (highest).
**SDT^a^-based variables**			
	Intrinsic motivation	Numerical; motivation	Average score of the intrinsic motivation items on the scale: a decimal number between 1 (lowest) and 5 (highest).
	Identified extrinsic motivation	Numerical; motivation	Average score of the identified extrinsic motivation items on the scale: a decimal number between 1 (lowest) and 5 (highest).
	Introjected extrinsic motivation	Numerical; motivation	Average score of the introjected extrinsic motivation items on the scale: a decimal number between 1 (lowest) and 5 (highest).
**Outcome variables**			
	Retention	Numerical; training	Measured as the total number of days from the user registration date in the app to their last recorded training session.
	Frequency, weekly average	Numerical; training	Calculated by dividing the total number of user sessions by the total number of weeks between their first and last recorded sessions.
	Adherence	Percentage; training	Defined as the percentage alignment between the user’s actual weekly training frequency and their predefined weekly training goal.

^a^SDT: self-determination theory.

Motivational data, which had been previously collected (March 20, 2022) by means of an ad-hoc scale (Table S2 in Multimedia Appendix 1), provided insight as to the motivational regulation of a subsample (n=753) of MH users. The scale was based on the self-determination theory (SDT) [19,20]. It showed good fit indices and a 3-factor structure as confirmed per exploratory and confirmatory factor analyses, with internal consistency indices >0.830 for the 3 subscales (intrinsic, identified extrinsic, and introjected extrinsic motivations). Data obtained through surveying (n=753) and data obtained from the MH fitness app (n=2771) were then merged, and a sample consisting of n=328, for which both training and motivational data were available, was obtained. Thirty-nine registries had to be disregarded due to missing data for the calculation of adherence and weekly training frequency outcome variables. A resulting total of 289 was complete for all explanatory and outcome study variables.

### Descriptive Analysis for Sociodemographic, Training, and Motivation Variables

Following data cleaning, descriptive statistics were computed to summarize and describe the dataset’s characteristics. Frequencies and percentages were calculated for categorical variables to provide an overview of their distribution and proportions within the sample. For numerical variables, measures of central tendency (mean and median) and measures of dispersion (minimum, maximum, and quartiles) were obtained to characterize data distribution and its variability.

### Inferential Analysis of Explanatory Variables

Normality was assessed using the D’Agostino-Pearson test, along with skewness and kurtosis coefficients to quantify distributional properties. Additionally, histograms and quantile-quantile plots were inspected to visually evaluate deviations from normality. The results indicated significant departures from normality, and nonparametric tests were used for subsequent analyses. The Kruskal-Wallis test was conducted to evaluate differences among groups, followed by post-hoc analysis using the Bonferroni correction to adjust for multiple comparisons. The effect size was assessed using Dunn, which quantifies the magnitude of observed differences. To examine relationships between numeric variables, Spearman correlation tests were performed. The Holm correction was applied to control for multiple comparisons and to adjust the significance levels accordingly. For training behavior analysis, the categorical variables evaluated were sex, Pro version, self-declared level of previous PA, body type, fitness goal, and training schedule. Additionally, explanatory numerical variables included age, BMI, subjective body fat, perceived difficulty, and enjoyment. Intrinsic, identified extrinsic, and introjected extrinsic motivations were also considered (Table 1). The relationship of all these variables was analyzed with three outcome variables: adherence, frequency, and retention.

### Inferential Analysis of Outcome Variables

Adherence was calculated as the percentage match between the target weekly frequency, as selected by the user upon sign-up, and the actual, executed weekly training frequency. The latter was averaged by dividing the total number of executed sessions by the total number of weeks from the sign-up date to the last executed session for the given user. Finally, retention was measured as the total number of days from the user sign-up date to the user’s last recorded training session (Table 1). Outcome variables were analyzed through the same procedures as explanatory variables.

### Ethical Considerations

Ethical approval for this study was obtained from the Research Ethics Committee of Universitat Ramon Llull in March 2020 (reference code 1920003P). All included users provided informed consent to use their data for research purposes, either through the app at registration or through the motivational survey. All data were anonymized before analysis, ensuring the privacy and confidentiality of participants in compliance with data protection regulations. No compensation was provided to participants, as the data were collected retrospectively and only for research purposes.

## Results

### Descriptive Results for Sociodemographic, Training, and Motivation Variables

Our sample consisted of 2771 MH users. Of them, a 64.8% majority identified as male, and 35.2% as female. Their age range spanned from 21 to 64 years, with a median age of 43 years and a mean age of 42.45 years. Users’ fitness goals varied, with the largest segment (46.6%) aiming to “gain muscle” mass, followed by those wanting to “lose weight” at 32%. A smaller portion, 21.4%, pursued “antiaging” benefits. Table 2 provides full details on sample description and other results.

**Table 2:** Descriptive characteristics of the Mammoth Hunters user sample (N=2771).

Variable				Values
**Sex, n (%)**				
	Male		1796 (64.8)	
	Female		975 (35.2)	
**Age (years)**				
	Mean (SD)		42.45 (9.8)	
	Median (IQR)		43 (21)	
	Range (minimum-maximum)		21-64	
**Body type, n (%)**				
	Thin		1269 (45.8)	
	Midbuild		1419 (51.2)	
	Strong		191 (6.9)	
**Body fat**				
	Mean (SD)		22.2 (6.4)	
	Median (IQR)		20 (14)	
	Range (minimum-maximum)		6-40	
**BMI**				
	Mean (SD)		23.46 (2.9)	
	Median (IQR)		23.44 (3.8)	
**Subscription type, n (%)**				
	Pro (paid) users		1726 (62.3)	
	Standard (free) users		768 (27.7)	
**Physical activity level, n (%)**				
	Active		1640 (59.2)	
	Very active		454 (16.4)	
	Inactive		675 (24.4)	
**Actual training schedule, n (%)**				
	Morning		928 (33.5)	
	Afternoon		1372 (49.5)	
	Night		471 (17)	
**Training difficulty (1-10)**				
	Mean (SD)		5.56 (1.78)	
	Median (IQR)		5.40 (2.10)	
	Range (minimum-maximum)		1-9.5	
**Enjoyment (1-5)**				
	Mean (SD)		3.58 (0.82)	
	Median (IQR)		3.50 (1.20)	
**Motivation (1-5)**				
	**Intrinsic**			
		Mean (SD)	4.01 (0.74)	
		Median (IQR)	4 (0.90)	
		Range (minimum-maximum)	1.5-5	
	**Identified extrinsic**			
		Mean (SD)	4.42 (0.50)	
		Median (IQR)	4.67 (0.67)	
		Range (minimum-maximum)	3-5	
	**Introjected extrinsic**			
		Mean (SD)	4.22 (0.61)	
		Median (IQR)	4.33 (1)	
		Range (minimum-maximum)	1-5	
**Retention (days)**				
	Mean (SD)		125.99 (92.60)	
	Median (IQR)		132.72 (144.36)	
	Range (minimum-maximum)		3.49-410.82	
**Training frequency (sessions per week)**				
	Mean (SD)		1.87 (1.52)	
	Median (IQR)		1.62 (1.84)	
	Range (minimum-maximum)		0.07-6.59	
**Adherence** (%)				
	Mean (SD)		54.24 (32.81)	
	Median (IQR)		47.31 (52.81)	
	Range (minimum-maximum)		1.18-166.67	

**Figure 1:**
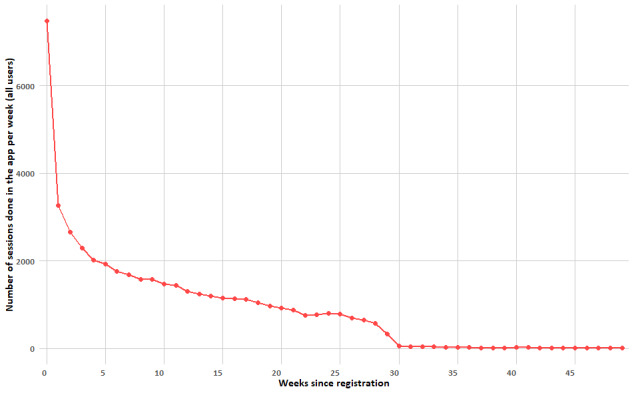
Total number of sessions in time (per training week) for all users (N=2771).

Figure 1 illustrates the number of training sessions completed each week, by the total number of users, over a 49-week span. At the beginning, there was a sharp peak in the number of training sessions, with 7469 sessions recorded in the first week after user registration, for a total of 2771 users. Following this peak, the number of sessions decreased rapidly over the next several weeks. It declined to 2295 by the end of the first month (a reduction of 69.3%), to 1678 by the end of the second month, and to 1448 (a reduction of 80.6%) by the end of the third. By around week 10, the decline began to stabilize, though a gradual downward trend persisted. By the 30th week, the number of sessions plateaued at a much lower level, approximately below 100 sessions in a week.

The time from user registration to their first training session ranged from 0 to 319.84 days, with a median delay of 7.98 days and a mean of 30 days. Most users initiated training within the first few days after registering, and the frequency declined steeply after 10 days. Delays beyond 50 days were rare, and only a very small proportion of users waited more than 100 days. A small subgroup of 31 users showed exceptionally long delays between 200 and 350 days.

### Differences Between Training Outcome Variables

#### Retention Results

**Figure 2:**
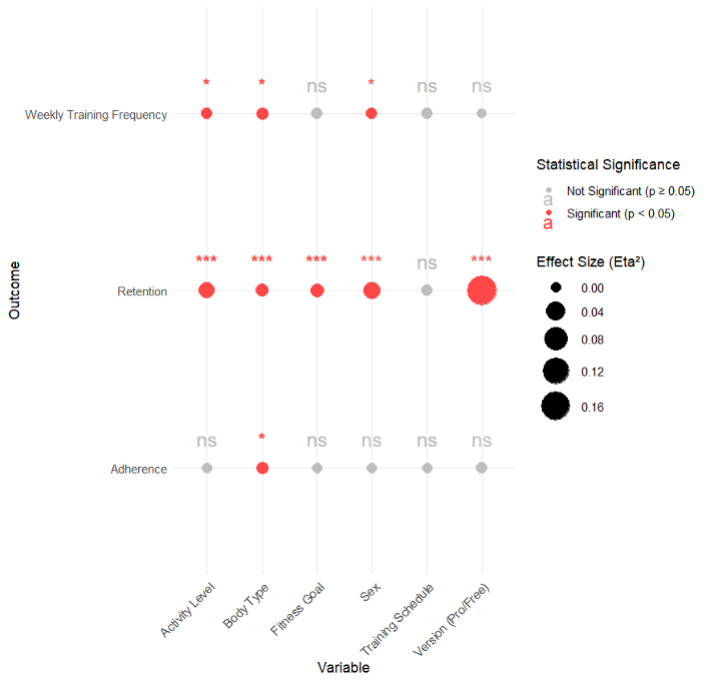
Summary of Kruskal-Wallis findings for each outcome variable.

All categorical variables except for training schedule showed statistically significant differences to retention (Table 3). For the sex variable, retention values were statistically significantly (*P<*.001) higher in the “male” group when compared to the “female” group, though the effect size was small (Dunn *r=*0.14). For the Pro version variable, indicative of whether the user was or was not subscribed for service at the time of data download, retention values were statistically significant (*P*<.001) with a moderate-to-large effect size (Dunn *r*=0.41), higher in the “yes” group than in the “no” group.” In regard to activity level, retention values were statistically significant (*P<*.001, Dunn *r=*0.11; and *P<*.001, Dunn *r=*0.07, respectively) and were higher for the “active” and “very active” groups than for the “inactive” group. No statistically significant (*P*>.05, Dunn *r=*0.03) differences were found in retention values between the “active” and “very active” groups. Refer to Figure 2 for further details on the Kruskal-Wallis results for the outcome variables.

**Table 3:** Retention (in days), relative to categorical variables levels according to the multivariate analysis of variance (n = 2771).

Variable and group		Mean (SD)	H^a^	*η²* ^b^	*P* ^c^
**Sex**			56.151	0.019	<.001^d^
	Female	109.43 (75.51)			
	Male	134.99 (73.86)			
**Pro version**			459.965	0.163	<.001^d^
	No	80.73 (70.11)			
	Yes	153.53 (64.47)			
**Activity level**			47.414	0.016	<.001^d^
	Inactive	109.74 (74.84)			
	Active	130.69 (75.65)			
	Very active	139.72 (69.57)			
**Body type**			14.303	0.004	.001^e^
	Mid	132.37 (74.16)			
	Thin	120.16 (76.27)			
	Strong	117.44 (75.63)			
**Fitness goal**			21.982	0.007	<.001^d^
	Antiaging	128.45 (70.23)			
	Gain	132.15 (76.62)			
	Lose	115.8 (75.18)			
**Training schedule**			3.613	0.000	.16
	Afternoon	125.51 (74.23)			
	Morning	128.49 (76.54)			
	Night	119.07 (77.71)			

^a^H: Kruskal-Wallis H value.

^b^*η*2: eta squared.

^c^*P:* Kruskal-Wallis significance.

^d^*P*<.001.

^e^*P*<.01***.***

For the body type variable, the “mid” group retention values were statistically significantly higher (*P<*.002; Dunn *r* 0.07) than those in the “thin” group. No statistically significant differences were found in retention values between the “mid” and “strong” groups, nor between the “strong” and “thin” groups (*P*>.05; Dunn *r=*0.04 and *P*>.05; Dunn *r=*0.00, respectively). In the fitness goal variable, retention values were significantly lower for the “lose weight” group compared to the “antiaging” and “gain muscle” groups (*P<*.02; Dunn *r=*0.05 and *P<*.001; Dunn *r=*0.09, respectively) (Table 4).

**Table 4:** Post-hoc comparison of retention by categorical variable levels.

Variable	Comparison	U^a^	*P* value
Sex	Female-male	–7.493	<.001b
Pro version	No-yes	–21.447	<.001b
Activity level	Active-inactive Active-very active Inactive-very active	6.067 –1.796 –5.641	<.001b .22 <001b
Body type	Mid-strong Mid-thin Strong-thin	2.034 3.579 –0.234	.13 <.001c >.99
Fitness goal	Antiaging-gain Antiaging-lose Gain-lose	–0.607 2.858 4.597	>.99 .01d <.001b
Training schedule	Afternoon-morning Afternoon-night Morning-night	–1.062 1.266 1.850	.87 .62 .19

^a^*U:* Standardized test statistic.

^b^***P*<.001**.

^c^*P*<.01.

^d^*P*<.05.

Regarding explanatory numerical variables, all of them showed statistically significant correlations with retention. Age had a moderate positive correlation (*r=*0.21; *P*<.001) with retention, while subjective body fat and BMI showed a low negative correlation (*r=*–0.13; *P*<.001; *r*=–0.06; *P*<.01, respectively). Training difficulty had a moderate, positive correlation (*r=*0.24; *P*<.001), and enjoyment had a low, positive correlation (*r=*0.11; *P*<.001).

Of all motivation dimensions, intrinsic motivation had the highest positive correlation (*r*=0.19; *P*<.01) with retention. Identified extrinsic motivation had a small, statistically significant, positive correlation (*r*=0.12; *P*<.05). Introjected extrinsic motivation had a much lower, positive, and nonstatistically significant correlation (*r=*0.07; *P*>.05; Table 5).

**Table 5:** Results of the correlation tests for motivation variables (N=289).

Variable			*r* ^a^		*P* value		
**Retention**							
	INTRINS^b^	0.19		.01^c^			
	IDENT, extr.^d^	0.12		<.05^c^			
	INTROJ, extr.^e^	0.07		.22			
**Frequency, weekly**							
	INTRINS	0.001		>.99			
	IDENT, extr.	0.008		>.99			
	INTROJ, extr.	0.168		.01^c^			
**Adherence**							
	INTRINS	–0.002		>.99			
	IDENT, ext.	0.021		>.99			
	INTROJ, ext.	0.138		.06			

^a^*r:* Spearman correlation coefficient.

^b^INTRINS: Intrinsic motivation.

^c^*P*<.05.

^d^IDENT, extr.: Identified extrinsic motivation.

^e^INTROJ, extr.: Introjected extrinsic motivation.

#### Average Weekly Frequency Results

Weekly frequency was found to be statistically significantly associated with sex, activity level, and body type (*P<*.05) and not significantly related to the Pro version, fitness goal, or training schedule (Table 6).

**Table 6:** Average weekly training frequency relative to categorical variables levels. Multivariate analysis of variance (N=2771).

Variable		Mean (SD)	H^a^	*η²* ^b^	*P* ^c^
**Sex**			3.950	0.001	.04^d^
	Female	1.82 (1.35)			
	Male	1.9 (1.29)			
**Pro version**			0.122	-0.001	.78
	No	1.94 (1.45)			
	Yes	1.83 (1.21)			
**Activity level**			6.199	0.001	.04^d^
	Inactive	1.83 (1.34)			
	Active	1.85 (1.29)			
	Very active	2.05 (1.31)			
**Body type**			8.324	0.002	.02^d^
	Mid	1.77 (1.23)			
	Thin	1.96 (1.37)			
	Strong	2.02 (1.39)			
**Fitness goal**			4.363	<0.001	.11
	Antiaging	1.79 (1.28)			
	Gain	1.91 (1.28)			
	Lose	1.85 (1.36)			
**Training schedule**			4.519	0.001	.10
	Afternoon	1.82 (1.30)			
	Morning	1.93 (1.32)			
	Night	1.94 (1.30)			

^a^H: Kruskal-Wallis H value.

^b^*η2*: eta squared.

^c^*P*: Kruskal-Wallis significance.

^d^*P*<.05.

For the sex variable, figures in the “male” group were statistically significant (*P<*.005; Dunn *r=*0.04) and higher than those for the “female” group. In the activity level variable, the “very active” group values were significantly higher (*P*<.05; Dunn *r=*0.05) than those in the “inactive” group. Neither the “active” versus “inactive” groups nor the “active” versus “very active” groups showed any statistically significant differences in weekly training frequency values. For the body type variable, values in the “thin” group were statistically significantly (*P<*.04; Dunn *r=*0.05) higher than those of the “mid” group. No statistically significant differences were found between the “mid” and “strong” groups, nor between the “strong” and “thin” groups, for weekly training frequency values. For more detail on multivariate analyses of variance results for weekly training frequency, please refer to Table 7.

**Table:** Post-hoc comparison of average weekly training frequency by categorical variable levels.

Variable	Comparison	*U* ^a^	*P* value
Sex	Female-male	–1.988	.04b
Pro version	No-yes	0.349	.73
Activity level	Active-inactive Active-very active Inactive-very active	0.599 –2.218 –2.427	>.99 .08 .04b
Body type	Mid-strong Mid-thin Strong-thin	–1.989 –2.501 0.727	.14 .04b >.99
Fitness goal	Antiaging-gain Antiaging-lose Gain-lose	–1.716 –0.372 1.679	.26 >.99 .28
Training schedule	Afternoon-morning Afternoon-night Morning-night	–1.824 –1.508 –0.379	.20 .40 >.99

^a^*U* = standardized test statistic.

^b^*P*<.05.

After corrections for multiple comparisons, none of the explanatory numerical variables reflected statistically significant correlations with weekly training frequency.

Regarding types of motivation, only introjected extrinsic motivation correlated significantly with training frequency *(r*=0.17; *P*<.05), after corrections for multiple comparisons, with a moderate-low positive correlation. Intrinsic and identified extrinsic motivations showed close to no correlation with frequency (*r=*0.00 and *P*>.05 for both) (Table 5).

#### Adherence Results

Adherence was found to be associated with one of the categorical variables (Table 8). These differences were found in body type, which presented a highly statistically significant (*P<*.005; Dunn *r=*–0.06) difference between the groups “mid” and “thin”. In particular, the values for the “mid” group were statistically significantly lower than those for the “thin” group. No statistically significant differences were found between groups “mid” and “strong,” nor between “strong” and “thin,” in adherence to the training program.

**Table 8:** Adherence, relative to categorical variables levels. Multivariate analysis of variance (N=2771).

Variable and group			Mean (SD)		H^a^		*η²* ^*b*^		*P* ^c^
**Sex**					1.479		0.000		.22
	Female	54.02 (38.07)							
	Male	54.35 (34.88)							
**Pro version**					3.260		0.000		.07
	No	57.35 (39.85)							
	Yes	52.35 (33.37)							
**Activity level**					2.394		0.000		.30
	Inactive	53.76 (38.29)							
	Active	53.91 (35.09)							
	Very active	57.01 (35.39)							
**Body type**					7.322		0.002		.03^d^
	Mid	52.1 (35.28)							
	Thin	56.96 (37.32)							
	Strong	52.01 (31.57)							
**Fitness goal**					2.960		0.000		.23
	Antiaging	52.52 (34.7)							
	Gain	55.34 (35.53)							
	Lose	53.48 (37.37)							
**Training schedule**					2.843		0.000		.24
	Afternoon	53.12 (35.66)							
	Morning	56.05 (36.95)							
	Night	53.61 (34.38)							

^a^*H*: Kruskal Wallis H value.

^b^*η2*: eta squared.

^c^*P:* Kruskal-Wallis significance.

^d^*P*<.05.

No statistically significant differences were found between adherence and any of the groups in any of the remaining categorical variables (Table 9).

**Table 9:** Post-hoc comparison of adherence by categorical variables levels.

Variable	Comparison	*U* ^a^	*P* value
Sex	Female-male	–1.216	.22
Pro version	No-yes	1.806	.07
Activity Level	Active-inactive Active-very active Inactive-very active	0.832 –1.082 –1.539	>.99 .84 .37
Body Type	Mid-strong Mid-thin Strong-thin	–0.517 –2.703 –0.836	>.99 .02b >.99
Fitness Goal	Antiaging-gain Antiaging-lose Gain-lose	–1.282 –0.096 1.499	.60 >.99 .40
Training schedule	Afternoon - Morning Afternoon - Night Morning - Night	–1.680 –0.558 0.453	.28 >.99 >.99

^a^*U*: Mann-Whitney standardized test statistic.

^b^*P*<.05.

For explanatory numerical variables, none reflected statistically significant correlations with adherence. Similarly, no types of motivation showed statistically significant correlations with adherence. Introjected extrinsic motivation rendered the highest (*r=* 0.14; *P*>.05) positive correlation, while intrinsic and identified extrinsic motivation showed little to no correlation (*r=*–0.00; *P*>.05, and *r=*0.02; *P*>.05, respectively; Table 5).

### Summary of All Findings From the Inferential Analysis

A summary of all findings from the inferential analysis is presented in Table 10.

**Table 10:** Summary of statistically significant findings of the post-hoc inferential analyses.

Variable	Retention	Weekly training frequency	Adherence
Sex	Male>female	Male>female	—^a^
Pro version	Yes>no	—	—
Activity level	Active>inactive Very active>inactive	Very active>inactive	—
Fitness goal	Antiaging>lose Gain>lose	—	—
Body type	Mid>thin	Thin>mid	Thin>mid
Training schedule	—	—	—
Age	Positive correlation	—	—
BMI	Negative correlation	—	—
Subjective body fat	Negative correlation	—	—
Difficulty	Positive correlation	—	—
Enjoyment	Positive correlation	—	—
Intrinsic motivation	Positive correlation	—	—
Identified extrinsic motivation	Positive correlation	—	—
Introjected extrinsic motivation	—	—	—

^a^Not applicable.

## Discussion

### Summary of Key Findings

This study examined training behaviors among users of the MH fitness app and identified key factors associated with training behavior. Variables including adherence and retention were evaluated, with the latter having shown greater relevance in users long-term maintenance of training behavior. The main findings pointed at paid subscription and intrinsic motivation as being the most determinant factors to user retention. Other variables that correlated with retention included sex, body type, BMI, and fitness goal. In contrast, adherence was only linked to body type, while training frequency varied slightly by sex, activity level, motivation, and body type.

This piece of research involved 2771 individuals and is possibly one of the largest existing cohort studies of fitness app users to date. Previous large cohorts include the Konstanz Life Study with 1236 users of either fitness or nutrition apps [21]. Some systematic reviews have covered samples of 3555 participants from a total of 22 interventions (n=833 in the largest single study) [8] or 1622 total participants from 6 different studies [7]. Our work possibly also covers the longest time duration (18 months). Previous research has been 2-24 weeks [7], up to 5 months [22], or even 6 months in some cases [8].

Our sample figures fall within the “expected” ranges for a fitness app that offers high-intensity training, delivered electronically. Results are also in line with the systematic review by Stecher et al [8], which included participants between 10.6 and 61.5 years of age and found a mean of 39.6 (SD 6.5) years. Participants in this study presented some features worth noting, which were probably specific to our sample population. The majority (75.6%) of them were previously “active” or “very active.” This was most likely due to the fact that all data registries were obtained from an app update (MH version 2.0) which, naturally, received many of its users from the previous version. This could also partially explain why 62% of our users were on the Pro version (paid subscription). MH always offered a free training program upon first registration, so the newest users would be expected to be on a free deal, while more experienced users would naturally progress to payment modes.

The studied sample primarily pursued “muscle gain” or “weight loss” fitness goals. A remarkably small (21.4%) percentage trained for “antiaging” purposes. We initially interpreted this finding as a sign that individuals were focusing mainly on “appearance,” but this would have to be further investigated, as muscle gain [23,24], as well as weight loss [25], are also known markers of improved health [25] and consequently better aging.

### Attrition Rates and Perceived Difficulty

It is well-established that attrition rates in mobile apps are extremely high. Meyerowitz-Katz et al [26], in their 2020 meta-analysis, stated that up to 98% of people only use apps for a short period of time. Our results fully align with this marked tendency, as we appreciated a remarkable drop in the number of training sessions within the first few weeks of enrollment. There was an observable decline of 69.3% by the end of the first month, a reduction of 77.5% by the end of month 2, and an 80.6% decline by the end of month 3. These figures strike even harder if we assume that many enrollments allegedly came from MH users who were transitioning from the old to the new version of the app. Participants in our study preferred “afternoon” (12:31-20:00 hours) training sessions and declared mean rates of session “difficulty” of 5.56, over a total of 10 points. The “difficulty” variable and its results need to be interpreted with caution. In our study, “difficulty” was an equivalent of perceived exertion, and it aimed to be indicative of how hard the session had felt to the user. However, this data were inquired once the user had not only finished the training but also finished the cool-down phase, and this could have led to respondents underrating the perceived exertion derived from the main block of training. Contrary to our expectations, difficulty in our sample showed a strong positive correlation to retention, which could be interpreted as a sign that challenge fosters engagement. Indeed, there is previous evidence that complex, vigorous, or hybrid activities correlate with intrinsic motivation [27], which commonly underlines activity retention. Regarding constructs of adherence, this finding could also reflect a self-selection bias, where more committed users are more likely to opt for challenging sessions, thus reinforcing their engagement over time.

### Factors Influencing Training Frequency

Frequency of training seemed not to be related to factors such as age, BMI, declared enjoyment or perceived difficulty, subscription vs nonsubscription, declared fitness goal, or preferred training schedule. However, statistically significant differences were observed based on sex, previous activity level, motivation, and body type. Frequency of training was greater in the introjected motivation group, in males, in the very active vs inactive, and in the thin vs mid groups. One could argue that the controlled and external regulation of introjected motivation could explain the increased frequency observed in this group. This would partially align with previous research that points to the primacy of extrinsic motivation in exercise contexts [28]. The fact that introjected motivation seems to encourage higher training frequencies but no longer retention or higher adherence might be indicative of an enthusiasm that is not sustained over time. As to the user’s previous activity level, while it seems logical that highly active individuals would train more often, this could be influenced by their prior engagement with the MH app or other forms of PA. If they were former MH users, their higher frequency could indicate loyalty, whereas if their activity stemmed from external sources, it is noteworthy that they also engaged frequently with the app. In contrast, inactive individuals may have felt overwhelmed by structured training. Previous research highlights differences in how beginners perceive social comparison and networking features in fitness apps, as well as how exercise proficiency affects adherence [29]. Additionally, attitudes toward PA significantly impact behavior, with Feng et al [30] showing that greater activity levels correspond to deeper integration and sustained engagement. Another possible explanation is that very active users may use more app features, enhancing their overall experience and leading to higher engagement [30]. Our results should, however, be interpreted with caution, since despite statistical significance, effect sizes were small to very small, which is indicative of them having limited practical implications.

### Reflections on Adherence to mHealth Training

In our study, adherence did not correlate with age, sex, previous level of activity, declared fitness goal, being on a free plan versus subscription mode, training schedule, perceived difficulty, or enjoyment in sessions.

Only one statistically significant difference was found for adherence, and it was for the “thin” group, which showed higher adherence than the “mid” group. Both frequency and adherence in this study were correlated with “thin” body type, but in both cases the effect size was small, so the association may not imply high practical impact. Notably, no motivation type proved to be more relevant for adherence, in spite of several authors having pointed to the more autonomous regulations of motivation leading to increased adherence and persistence [31]. Recent evidence confirms that maintaining physical activity remains challenging for healthy adults, with persistent individual-level barriers (ie, lack of motivation, attitudes, and concerns about physical changes) [32]. Adherence results in our study ranged from 1.2% to 166.7%, which was an impactfully wide range. It is important to note that intensity and duration data were not consistently available across users, which limited our ability to construct the adherence measure. Adherence in this study was based only on training frequency, a limitation that highlights the need for more standardized and comprehensive adherence metrics in future app-based exercise research. These results brought us to the following insights. Adherence is rather a measure of precise forecasting, as it basically depends on the ability to foresee future behavior. In that case, several personal characteristics may come into play, which have not been assessed in this study, such as the concept of self-efficacy, ambition, the ability to plan in advance, or the ability to pursue goals. Similarly, in healthy adults, psychological factors such as self-efficacy, enjoyment, and planning were significant predictors of long-term adherence to PA, emphasizing the relevance of individual motivational and behavioral traits in sustained engagement [33]. In addition, a study identified lack of time, motivation, and fatigue as frequent barriers to PA in healthy young adults, while enjoyment and social support emerged as consistent facilitators [34]. We found the lowest adherence rates for those who trained 6 times per week, while the highest adherence values were for those who trained twice per week. Based on our results, individuals with lower frequency expectations managed better to fulfill their target plan and were, consequently, more adherent. Again, we see a disadvantage in how different researchers seem to measure and define adherence, in addition to the fact that electronically delivered interventions often lack a detailed reporting of it [35]. We note that in our study, adherence was based on training frequency (% of targeted versus actual), which naturally correlates both variables. In contrast, retention and adherence operate on different parameters, especially when the exercise program is nonprescribed, lacks external obligation, and has no set duration.

### Retention as a Key Variable, Distinct From Adherence

In this study, retention correlated significantly with most study variables. There was higher retention in the male group, in subscribers, in “active” and “very active,” in “mid” body types versus “thin,” and also higher retention when the fitness goal was “antiaging” or “gain muscle” versus “lose weight.” “Pro version” users exhibited higher retention, aligning with previous research linking price to commitment [29,36,37], suggesting that subscription may indicate greater interest. The effect size for our finding was moderate-to-large, which points at subscription possibly being the most determinant factor in long-term training behavior. All other correlations had small to very small effect sizes. “Thin” body types, which had shown correlation with frequency and adherence, did not display higher retention, potentially reflecting an initial enthusiasm that wanes over time. The finding that “antiaging” goals led to higher retention than “lose weight” aligns with theories suggesting that health-oriented goals promote sustained engagement. However, the fact that “gain muscle” goals also outperformed “lose weight” in retention suggests that aesthetic-driven objectives may still play a role in long-term engagement, challenging this interpretation.

The study found that only intrinsic motivation had a statistically significant positive correlation with retention, while no such correlation was observed between intrinsic motivation and adherence. The distinction between retention and adherence is emphasized, as these concepts are considered distinct. In line with previous evidence [28,38-40], our study reflects that intrinsic and identified autonomously regulated motivations are the strongest correlated with retention. There is, however, previous evidence that points at extrinsic regulations of motivation as possibly the most important ones for exercise contexts [28,41]. We agree with Wilson’s statement that future research with larger sample sizes is recommended, considering potential variations in extrinsic motivation types [28] and a revision of the commonly accepted theory that intrinsic motivation is the most desirable to engage in and sustain exercise activities.

To translate our findings into practical applications, we suggest that fitness app developers consider tailoring features to specific user subgroups (ie, providing targeted support or content adaptations for users with a “mid” body type). Moreover, including motivational aspects that support intrinsic regulation, such as goal-tracking tools, personalized feedback, and autonomy-enhancing design, may further increase retention and adherence.

### Strengths and Limitations

The present study focused on analyzing user training behavior by means of a cross-sectional study conducted on 2771 MH app users over a period of 18 months. To the authors’ knowledge, the largest study previously available was a cohort study conducted under the Konstanz Life Study, which followed a total of 1236 users of either fitness or nutrition apps [21]. Other revisions involved larger samples, such as that by Stecher et al [8], (with 3555 participants across 22 interventions) or He et al [7] (with 1622 participants from 6 studies). Previous studies followed participants for periods of 2 to 24 months [7,8,22]. Based on this evidence, our study could be the largest of its kind in sample size and follow-up period so far. Nonetheless, we acknowledge that broader meta-analyses may include larger cumulative samples and aggregated durations across multiple interventions and apps [26].

The use of real-world app data, combined with motivational surveys, provides valuable insights into user behavior, retention, and adherence patterns. Additionally, the study uses robust statistical analyses, including nonparametric tests and multiple correction methods, ensuring the reliability of the findings.

However, the research also has limitations. The cross-sectional design prevents establishing causal relationships between motivation, training behavior, and adherence. The dataset is limited to users of a single fitness app (MH), potentially restricting generalizability to other platforms with different features or user demographics. We obtained informed consent from 5858 users. Of those registries, 2771 were complete and eligible for analysis. This loss should be acknowledged as the fact that we only managed to merge motivation and training data for a total of 289 participants, which limits the statistical power of our motivation-related analyses. Finally, adherence was measured in terms of training frequency, which may not fully capture engagement in app-based fitness programs, highlighting the need for more nuanced adherence metrics in future research.

### Conclusions

This study provides crucial insights into the exercise behavior and retention patterns of MH app users, highlighting key factors that influence user engagement. New insights are shared in regard to how motivation relates to training behavior with fitness apps. Clear differentiations are presented between adherence and retention, as conceptualized by the study authors. Fitness apps are a promising tool toward more active lifestyles, but we are yet lacking a sound understanding of related human behavior. Strategies such as gamification, goal-setting, or prompting are available to app developers to increase user engagement. However, longitudinal studies and mixed methods approaches are needed both to study causality and explore qualitative drivers to trainee behavior.

## Appendix

Multimedia Appendix 1 Complete description of the Mammoth Hunters dataset, including all variable categories and the full motivation survey used in the study.

## Abbreviations

MH: Mammoth Hunters
mHealth: mobile health
PA: physical activity
SDT: self-determination theory
WHO: World Health Organization

## Data Availability

The datasets analyzed during this study are available from the corresponding author on reasonable request.
