# Supplementary material for: Analysis of Training Behavior in Users of a Fitness App: Cross-Sectional Study
Source: JMIR Mhealth Uhealth. 2026 Jan 8;14:e72201. doi: 10.2196/72201 (PMC12828317; doi:10.2196/72201)
Supplement: Multimedia Appendix 1 [file mhealth_v14i1e72201_app1.docx]

## Table S1. Simplified list of variables in the Mammoth Hunters original dataset.

| Category: achievements | | | |
| --- | --- | --- | --- |
| - Id  - name_en  - points | - created_at  - updated_at  - achievment_type | - Icon_url  - congratulations_en  - congratulations_es  - description_en | - description_es  - name_es |
| Category: exercise_executions | | | |
| - Id  - exercise_id | - session_set_execution_id  - reps_executed | - execution_time  - order | - created_at  - updated_at |
| Category: exercise_sets | | | |
| - Id  - session_set_id  - exercise_id | - order  - created_at  - updated_at | - intensity_modificator (null)  - track_reps | - time_duration  - reps |
| Category: exercises | | | |
| - Id  - legacy_id  - deprecated (t/f)  - body_parts_focused  - muscles  - joints  - name_en  - execution_time  - coach_id  - Video | - created_at  - updated_at  - replacement_legacy_id  - family (null, zero, other #)  - met_multiplier  - video_female  - video_male  - harder_variation_id  - easier_variation_id  - description_en | - reps  - time  - sub_family  - description_es  - implement_variation_id  - name_es  - notes_en  - notes_es  - t1_min  - t1_max  - excluded | - test_correction  - thumbnail  - thumbnail_male  - thumbnail_female  - thumbnail_400  - thumbnail_400_male  - thumbnail_400_female  - test_equivalent_id |
| Category: products | | | |
| - Id  - price  - name  - store_reference  - currency | - local  - created_at  - updated_at | - store  - available  - has_trial  - trial_days | - discount_percentage  - discount_forever  - period |
| Category: profiles | | | |
| - Id  - Gender  - activity_level  - goal | - max_fat_level  - min_fat_level  - fat_level | - name | - created_at  - updated_at |
| Category: program_profiles | | | |
|  | - Id  - program_id  - profile_id | - created_at  - updated_at |  |
| Category: program_sessions | | | |
|  | - Id  - program_id  - session_id | - created_at  - updated_at |  |
| Category: programs | | | |
| - Id  - user_id  - pro  - available  - strength  - endurance | - technique  - flexibility  - intensity  - description_es  - auto_generated | - code_name  - next_program_id  - priority_order  - created_at | - updated_at  - description_en  - name_es  - name_en |
| Category: session_block_executions | | | |
| - Id  - session_execution_id  - order | - created_at  - updated_at | - block_type  - reps_executed  - execution_time |  |
| Category: session_blocks | | | |
| - Id  - sessionexecution_id  - order | - block_type  - loop | - time_duration  - created_at  - updated_at |  |
| Category: session_execution_summaries | | | |
| - Id  - session_execution_id  - total_reps  - total_time  - reps_per_min  - total_kcal  - reps_per_exercise | - mins_per_exercise  - reps_per_min_per_exercise  - reps_set_per_block  - time_set_per_block | - reps_min_set_block  - body_parts_spider  - effort  - points  - value_of_session | - created_at  - updated_at  - average_reps_min_set_per_block  - name |
| Category: session_executions | | | |
| - Id  - user_program_id  - difficulty_feedback  - enjoyment_feedback  - reps_executed | - session_id  - discarded  - discard_reason  - scheduled_at | - feedback_comment  - execution_time  - order  - created_at | - updated_at  - front_end_id  - imported |
| Category: session_set_executions | | | |
| - Id  - order | - session_block_execution_id  - reps_executed (null) | - execution_time (Null)  - created_at | - updated_at |
| Category: session_sets | | | |
| - Id  - order  - session_block_id | - loop  - Level (null) | - time_duration (null)  - reps (null)  - session_set_type (null) | - created_at  - updated_at |
| Category: sessions | | | |
| - Id  - order  - session_type  - time_duration  - code_name  - name_en | - name_es  - calories  - description_en  - warmup_id  - cooldown_id | - Level (null)  - reps (null)  - created_at  - updated_at  - strength (0-null)  - endurance (0-null) | - technique (0-null)  - flexibility (0-null)  - intensity (0-null)  - description_es |
| Category: subscriptions | | | |
| - Id  - user_id  - product_id  - program_id  - status  - cancelled  - store_metadata | - affiliate_code  - receipt_data  - created_at  - updated_at  - cancelled_at (null) | - Platform (nulls)  - transaction_body  - start_date  - end_date  - subscription_type | - offer_code  - cancellation_reason (programmation errors)  - receipt_data |
| Category: user_achievements | | | |
| - Id  - user_id | - achievement_id | - created_at  - updated_at |  |
| Category: user_programs | | | |
| - Id  - user_id  - program_id | - active  - enjoyment_notes | - current_session_id  - completed | - created_at  - updated_at  - enjoyment |
| Category: USERS | | | |
| - Id  - user_id  - created_at  - updated_at  - gender  - date_of_birth  - height  - weight  - activity_level  - goal  - body_type  - body_fat  - sign_in_count  - total_sessions  - total_time  - kcal_per_session  - reps_per_session | - workout_setting_voice_coach  - workout_setting_sound  - workout_setting_vibration  - workout_setting_mobility  - workout_setting_cardio_warmup  - workout_setting_countdown  - notifications_setting  - training_days_setting  - language  - country  - points  - best_weekly_streak  - current_weekly_streak  - affiliate_code_signup | - newsletter_subscription  - Email  - encrypted_password  - reset_password_token  - reset_password_sent_at  - remember_created_at  - is_admin  - names  - last_name  - current_sign_in_ip  - last_sign_in_ip  - recover_password_code  - recover_password_attempts  - facebook_uid  - google_uid  - scientific_data_usage  - stripe_id  - provider  - uid | - moengage_id  - mix_panel_id  - apple_id_token  - imported  - platform  - login_token  - login_token_generated_at  - current_sign_in_at  - last_sign_in_at  - t_1 everything  - t_2 everything  - warmup_setting  - warmup_session_id  - affiliate_code |
| Category: daily_reports | | | |
| - Id  - report_data | - created_at  - updated_at | - report_date |  |
| Category: exercise_implements | | | |
| - Id  - exercise_id | - implement_id | - created_at  - updated_at |  |
| Category: implements | | | |
| - Id  - name_en | - name_es | - created_at  - updated_at |  |
| Category: offers | | | |
| - Id  - offer_code |  | - created_at  - updated_at |  |
| Category: product_offers | | | |
| - Id  - offer_id | - product_id | - created_at  - updated_at | - code_name |
| Category: program_implements | | | |
| - Id  - program_id | - implement_id | - created_at  - updated_at |  |
| Category: user_implements | | | |
| - Id  - implement_id | - user_id | - created_at  - updated_at |  |

Table S2. Motivation survey as conducted in n=753 Mammoth Hunters users and indication of correlated motivation as per factorial analysis.

| ITEMS IN MOTIVATION SURVEY | INTR | IDE | INJT |
| --- | --- | --- | --- |
| 1 Whenever I do a high intensity session and end up feeling tired, this encourages me to continue doing sessions in the future |  |  |  |
| 2 This physical activity program helps me improve my physical appearance |  |  |  |
| 3 I enjoy reaching the goals and objectives set by the physical exercise program |  |  |  |
| 4 The physical exercise program allows me to prevent future injuries |  |  |  |
| 5 I enjoy every moment of my time that I dedicate to physical exercise |  |  |  |
| 6 I cannot imagine my life without practicing sports |  |  |  |
| 7 The physical exercise program allows me to stay within adequate body weight |  |  |  |
| 8 The physical exercise program provides me with improved image towards others |  |  |  |
| 9 The physical exercise program allows me to improve my health |  |  |  |
| 10 The physical exercise program will keep me from having health issues in the future |  |  |  |

*Note:* INT*R =* intrinsic motivation; IDE = identified extrinsic motivation; INJT = introjected extrinsic motivation.
